# High-Performance Piezoelectric Micro Diaphragm Hydrogen Sensor

**DOI:** 10.1021/acssensors.4c03069

**Published:** 2025-03-13

**Authors:** Jihang Liu, Doris Keh Ting Ng, Yul Koh, Subhranu Samanta, Weiguo Chen, Md Hazwani Khairy Md Husni, Merugu Srinivas, Qingxin Zhang, Fuu Ming Kai, Peter Hyun Kee Chang, Yao Zhu

**Affiliations:** † Institute of Microelectronics (IME), Agency for Science, Technology and Research (A*STAR), 2 Fusionopolis Way, Innovis #08-02, Singapore 138634, Republic of Singapore; ‡ National Metrology Centre (NMC), Agency for Science, Technology and Research (A*STAR), 8 Cleantech Loop, #01-20, Singapore 637145, Republic of Singapore

**Keywords:** piezoelectric micro diaphragm, palladium sensing layer, hydrogen sensor, resonant gas sensor, response stress-based

## Abstract

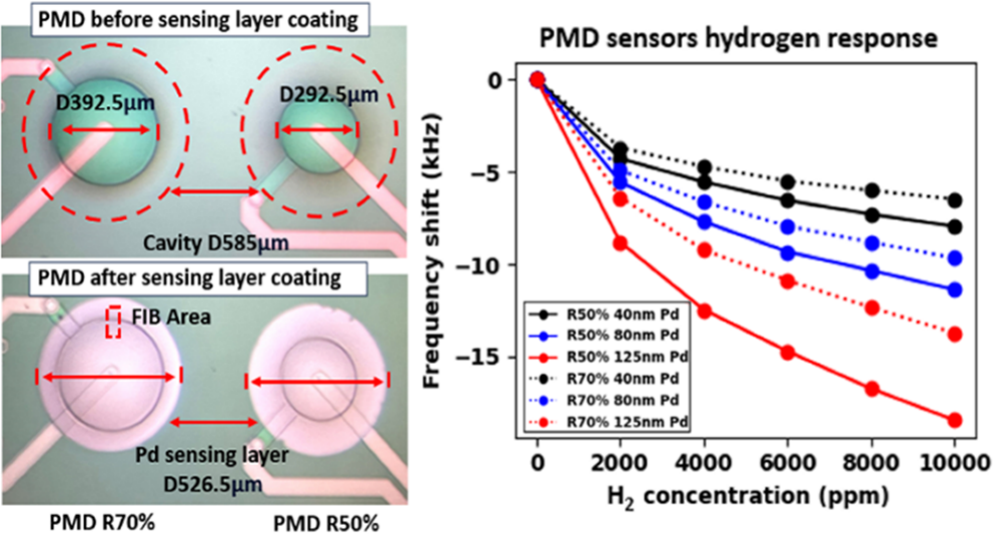

Highly sensitive, selective, and compact hydrogen (H_2_) sensors for safety and process monitoring are needed due to the growing adoption of H_2_ as a clean energy carrier. Current resonant frequency-based H_2_ sensors face a critical challenge in simultaneously achieving high sensitivity, low operating frequency, and miniaturization while maintaining a high figure of merit (FOM). This study addresses these challenges by introducing a novel piezoelectric micro diagram (PMD) H_2_ sensor that achieves an unprecedented FOM exceeding 10^4^. The sensor uniquely integrates a PMD resonator with a palladium (Pd) sensing layer, operating on a stress-based mechanism distinct from traditional mass-loading principles. Despite a low operating frequency of 150 kHz, the sensor demonstrates a remarkable sensitivity of 18.5 kHz/% H_2_. Comprehensive characterization also reveals a minimal cross-sensitivity to humidity and common gases and a compact form factor (600 μm lateral length) suitable for IC integration. The sensor’s performance was systematically evaluated across various Pd thicknesses (40–125 nm) and piezoelectric stack covering ratios (50% and 70%), revealing a trade-off between sensitivity and response time. This PMD H_2_ sensor represents a significant advancement in resonant frequency-based H_2_ sensing, offering superior sensitivity, compact size, and robust performance for diverse applications in H_2_ detection and monitoring.

The rising prominence of H_2_ as a clean energy source has heightened the demand for accurate H_2_ sensors, particularly for low-concentration leak detection. These sensors play a crucial role in safety applications across various industries, from automotive to aerospace.^1^ The ability to detect H_2_ at levels well below its lower flammable limit (4% in air) is essential for preventing potential hazards,^2^ especially in confined spaces where even small leaks can accumulate to dangerous concentrations. Consequently, the development of sensitive and reliable H_2_ sensors for low-concentration detection has become a priority impacting various aspects of modern life and technology.

Among various H_2_ sensing technologies, resonant frequency-based sensors offer several distinct advantages. These sensors exhibit high sensitivity and rapid response times,^3^ crucial for early leak detection. Unlike conventional electrical sensors, they are immune to electrical parasitic effects, enhancing reliability in diverse environments.^4^ Resonant frequency-based H_2_ sensors operate effectively at room temperature, eliminating the need for energy-intensive heating elements. This feature, combined with their inherently low power consumption, makes them ideal for portable and long-term monitoring applications.^5^ Furthermore, their potential for miniaturization aligns well with the trend toward compact, integrated sensing systems.^6^ These merits position resonant frequency-based H_2_ sensors as a promising solution for addressing the growing demand for efficient and reliable hydrogen detection across various sectors.

Resonant frequency-based H_2_ sensors consist of two main functional components: a resonator and a sensing layer. The resonator provides an electromechanical coupling signal with specific frequencies, while the sensing layer is responsible for H_2_ absorption and desorption. This design features high flexibility in various applications, with overall performance dependent on both components. Examples of resonators include film bulk acoustic resonators (FBAR), surface acoustic wave (SAW) devices, and quartz crystal microbalances (QCM), operating at frequencies ranging from GHz to MHz.^6−12^ Sensing layers typically utilize materials such as Pd, platinum (Pt), zinc oxide (ZnO), and its corresponding alloys or nanostructures.^7−15^

Recent studies have demonstrated the diverse capabilities of resonant frequency-based H_2_ sensors, highlighting their potential for various applications. FBAR H_2_ sensors excel in miniaturization and sensitivity. With a compact size of approximately 500 μm in lateral length, they achieve remarkable sensitivities: 5 MHz/% for 0–2% H_2_ detection using Pd layer at 2.2 GHz, and 6 MHz/% for 0–3% H_2_ detection using ZnO layer at 2.3 GHz.^7,8^ SAW sensors, while larger (>3000 μm lateral length), offer a range of sensitivities. These span from 1.5 kHz/% with a Pd/Cu layer at 150 MHz to 55 kHz/% with a Pd/ZnO layer at 129 MHz, both for 0–1% H_2_ detection.^9,10^ QCM sensors present an intermediate size (>1800 μm) and varied performance: from 2.5 × 10^–3^ kHz/% for 2–20% H_2_ detection using graphitic carbon nitride (g-C_3_N_4_) at 9 MHz, to 5.3 kHz/% for 0–0.025% H_2_ detection with Pd at 165 MHz.^11,12^

Despite the advancements in resonant frequency-based H_2_ sensors, they still face a key challenge in optimizing their FOM, defined as the ratio of sensitivity to eigenfrequency.^16,17^ While higher sensitivity improves detection resolution, it often requires increased eigenfrequency according to the Sauerbrey mass equation,^7−12^ which in turn demands more sophisticated interface circuits to mitigate noise issues. FBAR sensors currently lead with FOM levels of 10^3^, followed by SAW sensors at 10^2^, and QCM sensors at 10^1^. The field urgently requires sensors with higher FOM values to enhance sensitivity without compromising performance or increasing circuit complexity. This necessitates innovative approaches in sensor design and material selection to advance H_2_ detection capabilities.

This work introduces a novel H_2_ sensing approach aimed at achieving an unprecedented FOM of 1 × 10^4^ or higher. The key innovation lies in the unique integration of a PMD resonator with a Pd sensing layer, a combination unexplored in previous H_2_ sensor research. This design leverages the PMD’s flexible vibration mode, characterized by a low eigenfrequency (in hundred kHz range), and its exceptional frequency sensitivity to structural stress changes.^17,18^ This stress-based mechanism, fundamentally different from Sauerbrey mass-loading principle, when combined with Pd’s superior H_2_ absorption properties, promises to dramatically enhance the H_2_ sensor performance. The concept’s viability was verified through theoretical analysis, finite element method (FEM) simulations, and comprehensive experimental validation, including sensor structure characterizations, H_2_ response sensitivity/time tests, and cross-sensitivity analysis.

## Model and Experiment

### Sensor Design

**Figure 1:**
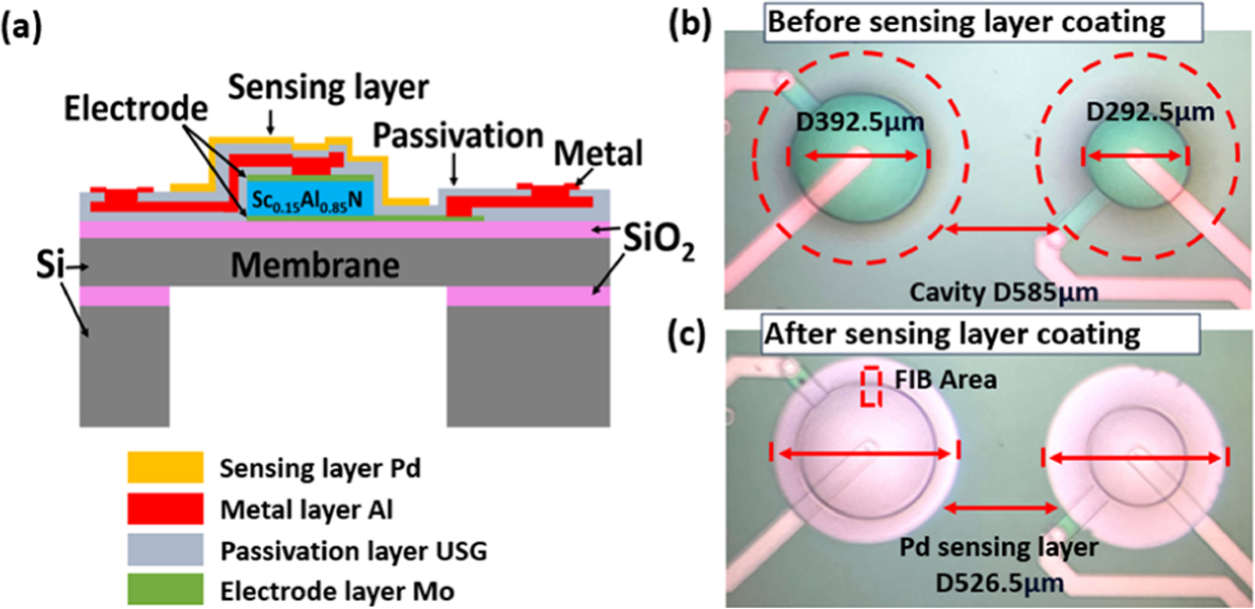
(a) Cross section view of PMD sensors. (b) Top view of PMD sensors before and (c) after the Pd coating.

The PMD sensor comprises two primary components: a bottom PMD resonator and a top Pd sensing layer, as illustrated in Figure 1a.

The PMD resonator’s structure is carefully engineered for optimal performance. It features a piezoelectric stack of 1-μm-thick Sc_0.15_Al_0.85_N, chosen for its minimal ferroelectric hysteresis and long-term piezoelectric stability,^19,20^ sandwiched between two 0.2-μm-thick molybdenum (Mo) electrodes. A 4-μm-thick epitaxial silicon elastic layer underneath enhances effective transverse driving efficiency by maintaining the neutral axis away from the piezoelectric stack,^21^ while providing appropriate structural flexibility for high response sensitivity. A 0.5-μm-thick silicon nitride (Si_3_N_4_) + undoped silicate glass (USG) isolation layer covers the piezoelectric stack for protection. The resonator is built on a patterned substrate with a 585 μm diameter etched through the cavity on the backside aimed for an ∼150 kHz resonant frequency. Electrical connectivity between the top (signal) and bottom (ground) electrodes is facilitated through vias and aluminum metal routing.

This study employs devices with 50% (R50%) and 70% (R70%) piezoelectric stack-to-cavity diameter covering ratios, as shown in Figure 1b,c respectively, both before and after Pd coating with a 90% diameter-to-cavity covering ratio. These design variations enable comprehensive analysis for precise H_2_ gas molecule detection while optimizing the PMD resonator’s performance.

### Sensor Working Principle

**Figure 2:**
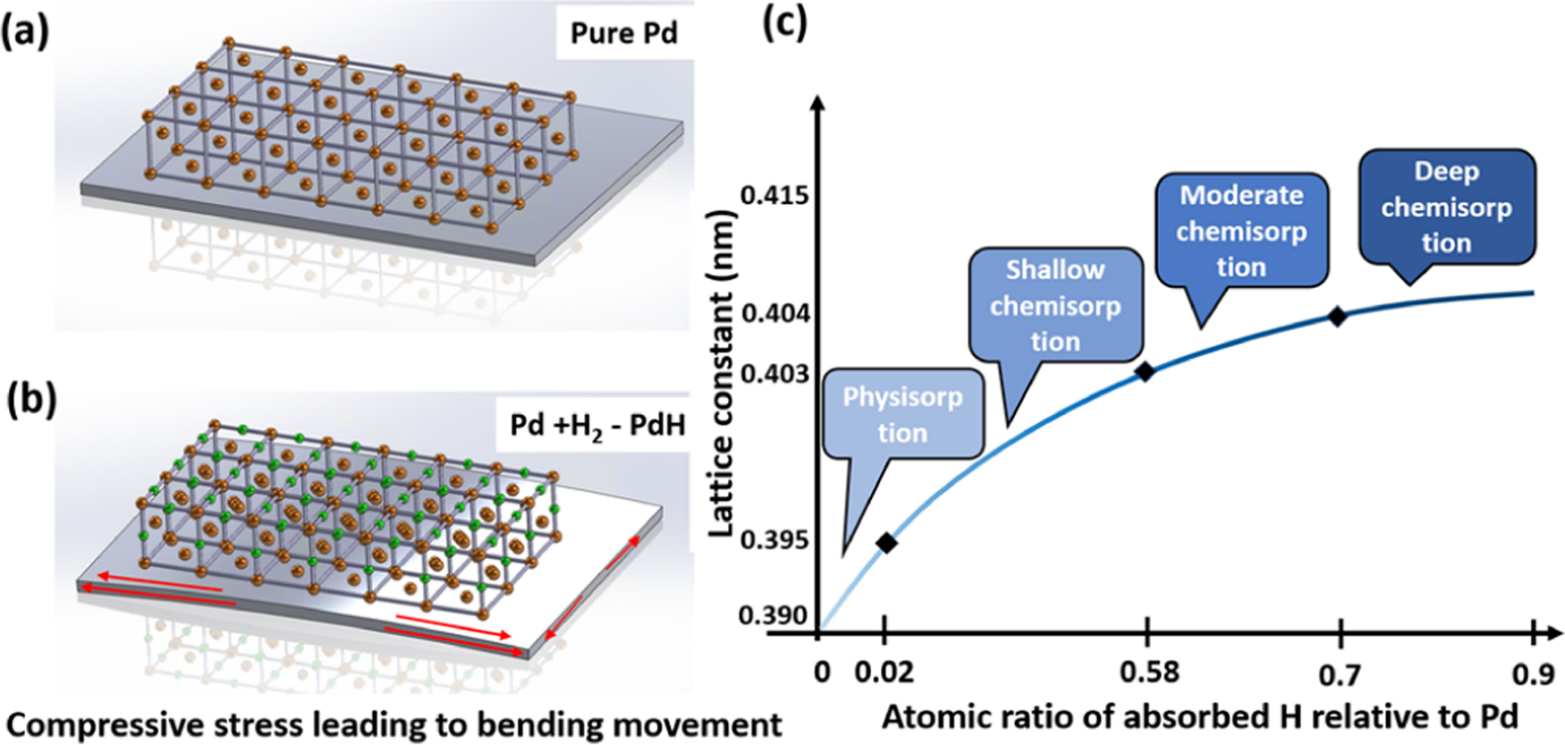
Schematics of Pd–H_2_ reaction progress: (a) Pure Pd film lattice attached on substrate, (b) PdH_*x*_ and Pd composite film lattice during H_2_ absorption, (c) PdH_*x*_ and Pd composite file lattice constant during H_2_ absorption.

The sensor’s working principle hinges on the Pd sensing layer’s interaction with ambient H_2_ molecules as depicted in Figure 2. This interaction disrupts interatomic forces within the Pd film,^22^ causing lattice expansion and inducing compressive stress that transforms the flat elastic diaphragm into a convex shape. The Pd–H_2_ interaction progresses dynamically from physisorption to deep chemisorption as H_2_ concentration increases, with 1% H_2_ potentially inducing ∼600 MPa of compressive stress in the 50 nm sensing film.^23^

Crucially, the PMD’s eigenfrequency is proportional to the square root of diaphragm stress as shown in eq 1.^24^ This relationship enables H_2_ concentration prediction through measured eigenfrequency shifts, effectively converting the H_2_ concentration into a detectable mechanical response and subsequent frequency change.
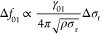
1where Δ*f*_01_ is the resonant frequency shift, γ_01_ is the flexible mode constant, σ_r_ is the average stress of the diaphragm, and ρ is the diaphragm density.

### Simulation

**Figure 3:**
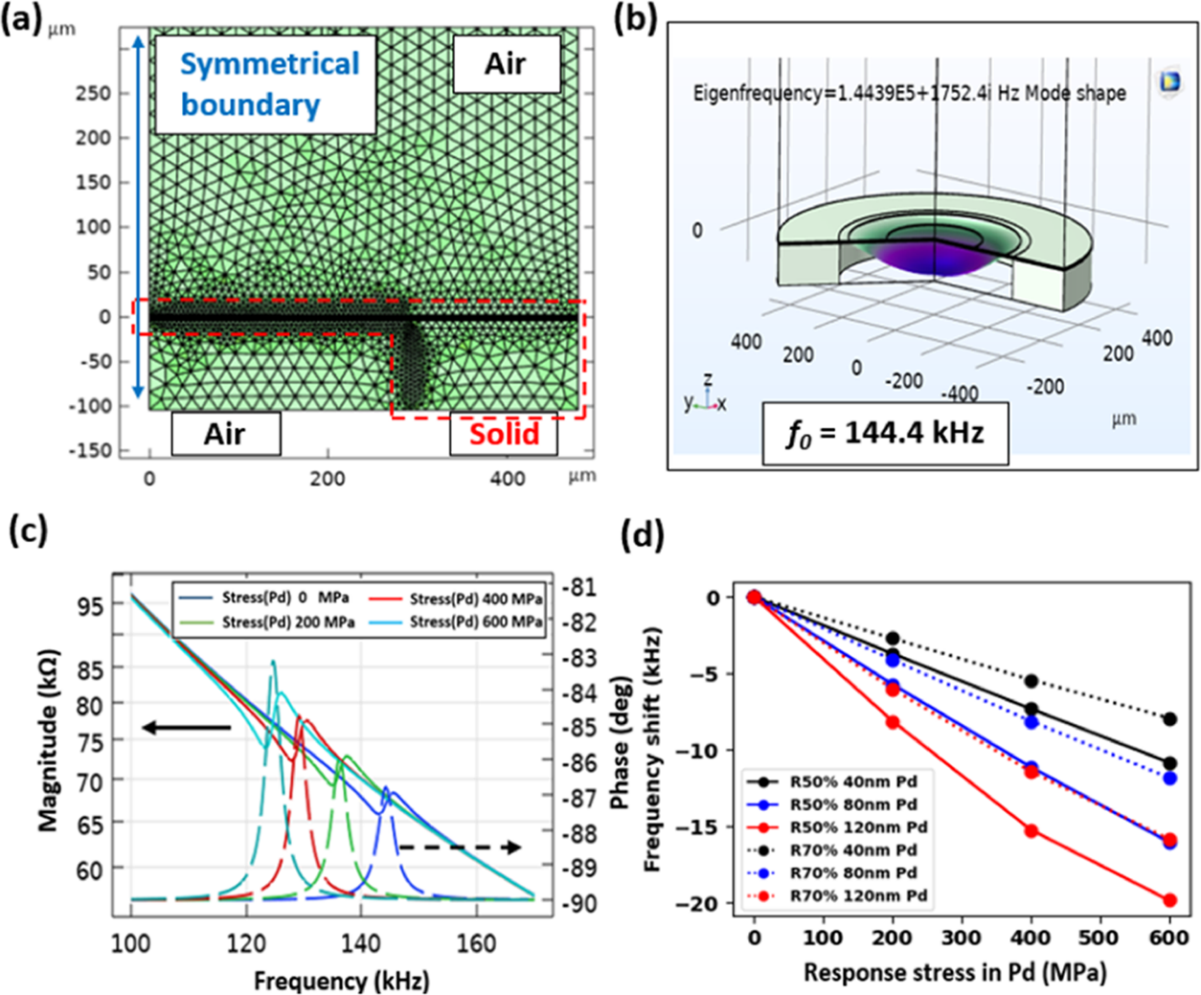
Simulation results of (a) meshing map and physic situation. (b) Eigenfrequency of PMD device and the mode shape. (c) Impedance magnitudes and phases change with compressive stress in Pd film for R70% 120 nm Pd coating device. (d) Frequency response of Pd stress change for PMD devices with various thicknesses of Pd coating.

Comprehensive COMSOL FEM simulations were conducted to theoretically analyze the PMD sensor’s resonance frequency response to sensing layer stress, as shown in Figure 3a. To optimize the computational efficiency, a 2D symmetrical model was employed, incorporating key interfaces of solid structures and surrounding acoustic media. The analysis combined eigenfrequency and frequency domain studies, ensuring consistency and validation. Figure 3b,c presents the critical findings: For example, a PMD device with 50% covering ratio and 120-nm-thick Pd coating (R50% 120 nm Pd) exhibited a 144.4 kHz resonant frequency under flexible vibration mode. Notably, as the Pd sensing layer’s response stress increases from 0 to 600 MPa, the device’s impedance resonant peak shifts from 144.4 to 124.2 kHz, demonstrating a clear correlation between stress and frequency response.

Figure 3d illustrates the comparative stress sweep simulations for various Pd sensing layer thicknesses (40, 80, and 120 nm) on both R50% and R70% devices. The results reveal a clear trend in sensitivity: the R50% device with a 120 nm Pd layer exhibited the highest sensitivity at 20.2 kHz per 600 MPa (equivalent to 20.2 kHz/% H_2_), while the R70% device with a 40 nm Pd layer showed the lowest sensitivity at 7.7 kHz per 600 MPa (7.7 kHz/% H_2_). This pattern suggests two key design principles for optimizing sensor sensitivity: (1) a smaller piezoelectric stack covering ratio and (2) a thicker Pd coating. These findings provide valuable guidance for enhancing the H_2_ response sensitivity potentially leading to more accurate H_2_ detection capabilities.

### H_2_ Test Setup

**Figure 4:**
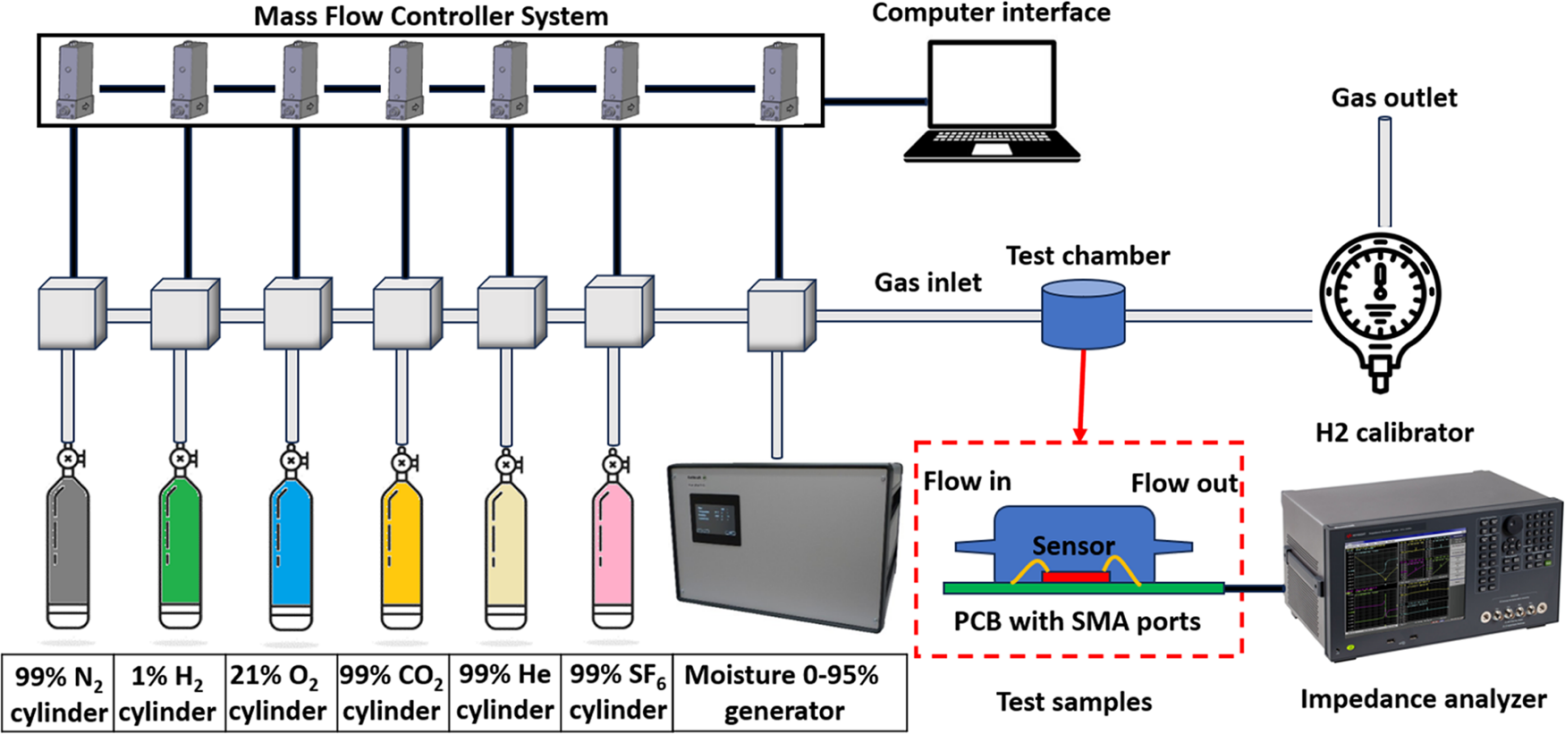
Experimental test setup schematic and related instruments.

The experimental test setup, displayed in Figure 4, comprises three main components: a gas control system, a test chamber, and data processing equipment. The gas control system features gas source cylinders (N_2_, H_2_, O_2_, CO_2_, He, and SF_6_), a Cellkraft Humidifier P-2 moisture generator, and corresponding mass flow meters controlled via computer interface software. This system enables precise gas combinations at a 1000 sccm flow rate for H_2_ response, moisture, and cross-sensitivity tests, with N_2_ serving as the default carrier gas. The test chamber consists of a 3D-printed plastic cap with gas inlet and outlet ports, attached to a PCB where the PMD sensor chip is mounted and wire-bonded for electrical connectivity. Throughout all experiments, an impedance analyzer measures the PMD sensor’s impedance spectrum, while a Model GPA2000 H_2_ calibrator ensures accurate H_2_ concentration calibration.

## Results and Discussion

### Sensor Structure Characterization

**Figure 5:**
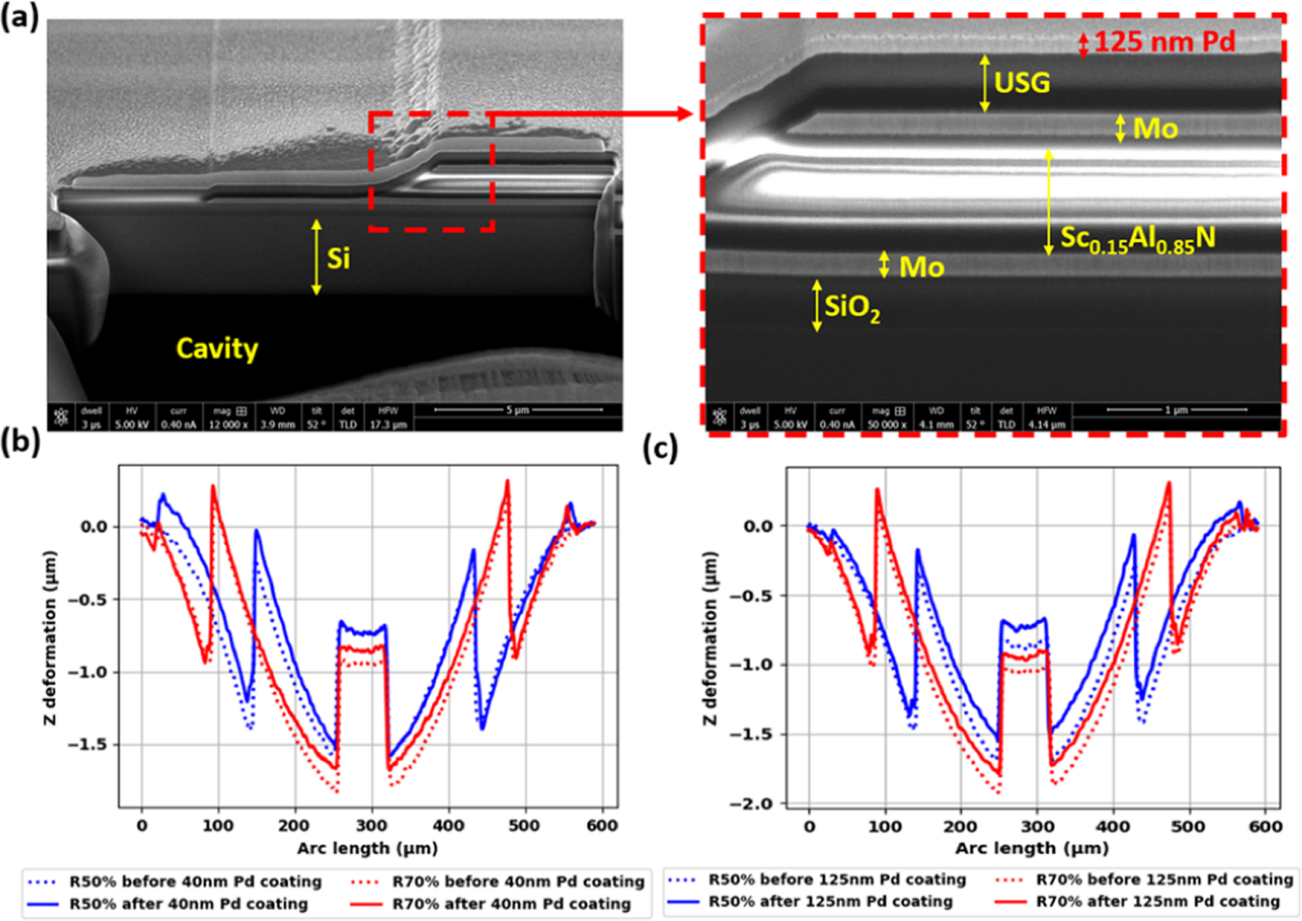
Pd film coating characterizations of (a) oblique angle SEM image after FIB cutting on the PMD electrode edge step area and its zoom-in view. (b) Optical profile of PMD sensors before and after 40 nm Pd coating. (c) Optical profile of PMD sensors before and after 125 nm Pd coating.

Pd film coating characterization was conducted using scanning electron microscopy (SEM) coupled with focused ion beam (FIB) cutting on the piezoelectric stack edge of devices with three different Pd thicknesses, as shown in Figure 5a. The measured Pd film thicknesses were 39.4, 84.6, and 125.0 nm, respectively, each within a ± 5.0 nm error range. Optical surface profiles before and after Pd coating for R50% and R70% devices, presented in Figure 5b,c, respectively, reveal crucial insights into the PMD devices’ structural characteristics and stress dynamics. Notably, R70% devices, with their larger piezoelectric stack covering ratio, exhibit approximately 0.4 μm greater concave deformation compared to R50% devices, indicating the dominance of tensile residual stress from the piezoelectric stack in determining initial deformation. The nearly identical initial states observed in devices with the same ratio underscore the excellent uniformity achieved in the fabrication process. Furthermore, the consistent deformation status before and after Pd coating for each device suggests that the addition of 40–125 nm pure Pd film does not significantly alter the devices’ mechanical stress state. These observations collectively highlight the robustness of the fabrication process and the minimal impact of Pd coating on the initial stress state, providing valuable understanding of the PMD devices’ behavior with varying Pd coating thicknesses.

### Sensor H_2_ Characterization

**Figure 6:**
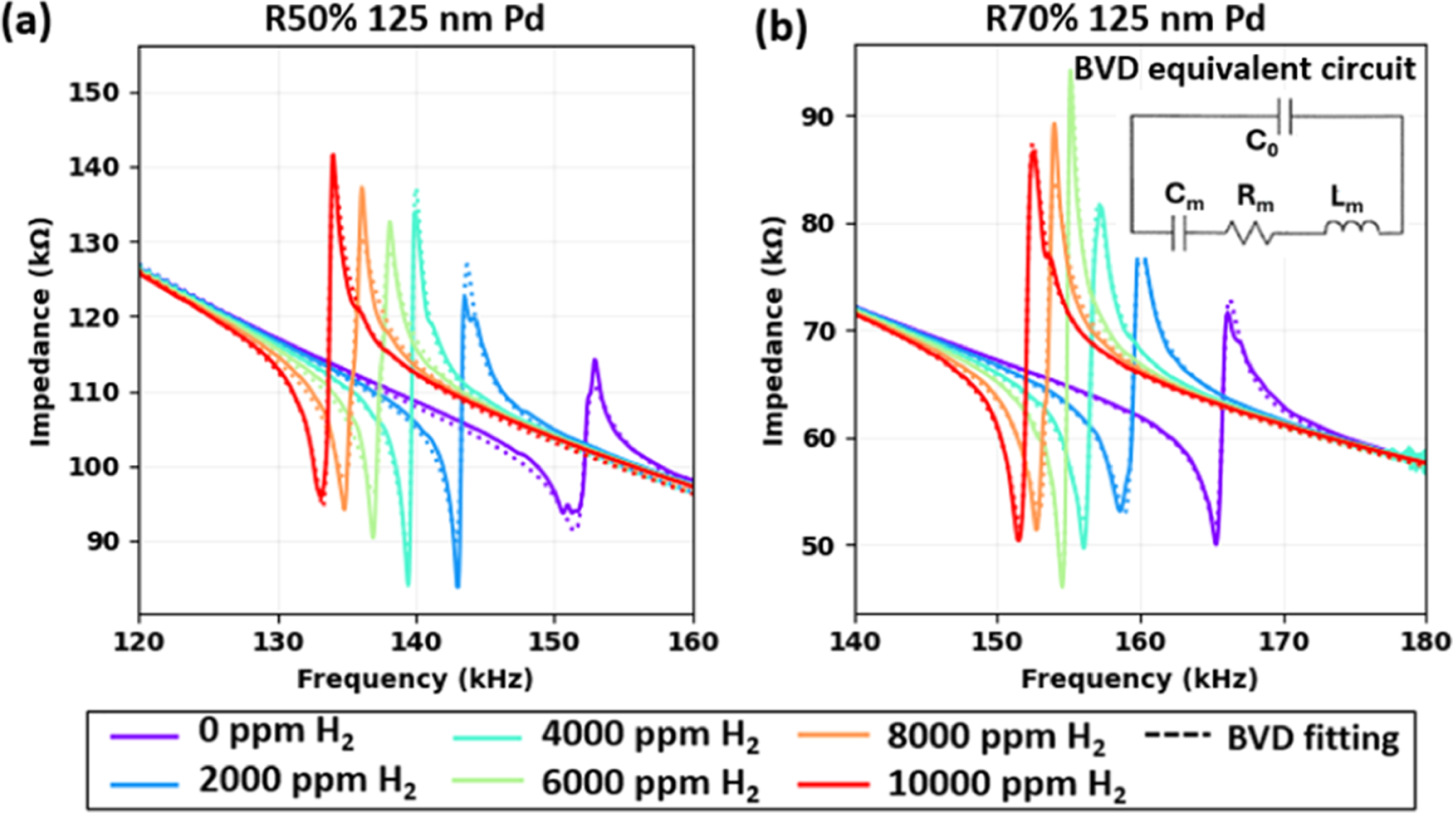
Impedance spectrum characterizations for (a) the R50% 125 nm Pd device and (b) the R70% 125 nm Pd device under various H_2_ concentrations and the corresponding BVD equivalent circuit fitting.

Impedance spectrum characterization of PMD sensors was conducted under H_2_ concentrations ranging from 0 to 10 000 ppm, with results for R50% and R70% devices (125 nm Pd coating) demonstrated in Figure 6a,b, respectively. As H_2_ concentration increases, both device types exhibit a leftward shift in resonant peak, with R50% devices showing a more pronounced shift compared with R70% devices. Notably, both device types display robust electrical signals across the full H_2_ response range, enabling accurate processing through a Butterworth-Van Dyke (BVD) equivalent circuit fitting. This fitting achieved excellent match quality (R^2^ > 99%), allowing for reliable extraction of key parameters, which are compiled in Table 1 for precise data analysis.

**Table 1:** BVD Equivalent Circuit Fitting Extraction Parameters for PMD R50% and R70% 125 nm Pd Sensors

	PMD R50% 125 nm Pd device							PMD R70% 125 nm Pd device						
H_2_ (ppm)	*f*_0_ (kHz)	*Q*	*K*_t_^2^ (%)	*C*_0_ (pF)	*C*_m_ (pF)	*R*_m_ (kΩ)	*L*_m_ (H)	*f*_0_ (kHz)	*Q*	*K*_t_^2^ (%)	*C*_0_ (pF)	*C*_m_ (pF)	*R*_m_ (kΩ)	*L*_m_ (H)
0	152.1	104.3	0.24	10.40	0.020	499.4	54.5	165.6	160.3	0.27	15.63	0.034	176.6	27.2
2000	143.3	185.2	0.23	10.39	0.020	303.9	62.5	159.4	141.3	0.34	15.57	0.043	163.4	23.1
4000	139.7	235.2	0.24	10.39	0.020	239.0	64.1	156.4	158.1	0.36	15.57	0.046	140.4	22.6
6000	137.4	116.2	0.32	10.39	0.027	370.8	49.9	154.8	268.2	0.33	15.58	0.041	93.3	25.7
8000	135.4	105.7	0.34	10.39	0.029	387.7	48.2	153.4	141.9	0.41	15.57	0.052	140.6	20.7
10 000	133.6	158.5	0.31	10.39	0.026	291.6	55.1	151.9	168.0	0.40	15.57	0.050	124.8	22.0

Analysis of the extracted key parameters in Table 1 reveals significant differences between PMD R50% and R70% devices with a 125 nm Pd coating. The R50% device, with its smaller piezoelectric stack covering ratio, exhibits a resonant frequency of 152.1 kHz, which is 13.5 kHz lower than the R70% device. This lower frequency is attributed to reduced stiffness and less impact from piezoelectric stack residual stress. Apparently, the R50% device demonstrates superior H_2_ sensitivity, with a frequency sensitivity of 18.5 kHz/%, 1.35 times higher than that of the R70% device. Conversely, the R70% device shows better electrical performance, with an average Q factor 15% higher and a K_t_^2^ value 25% higher than that of the R50% device. These findings highlight a trade-off between the smaller and larger piezoelectric stack devices: smaller piezoelectric stacks offer higher sensitivity but relatively lower electrical signal quality.

**Figure 7:**
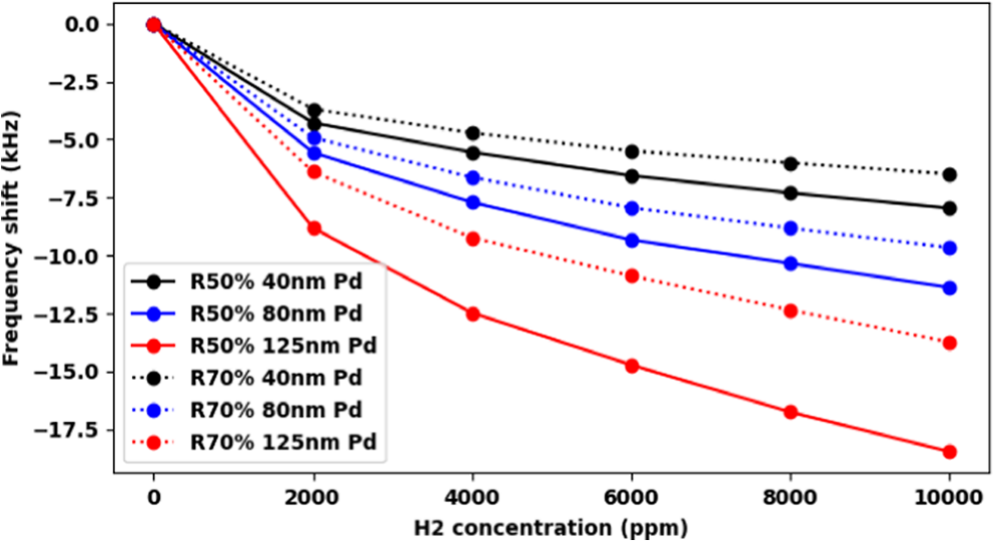
PMD H_2_ sensor sensitivity of R50% and R70% devices with 40–125 nm thickness of Pd coating.

The H_2_ sensitivities of PMD R50% and R70% devices with Pd coating thicknesses ranging from 40 to 125 nm are illustrated in Figure 7 revealing several key trends. For the same Pd thickness, R50% devices consistently demonstrate 1.2–1.35 times higher sensitivity than R70% devices, while increasing Pd coating thickness by a factor of 1 within the same piezoelectric stack ratio results in a 1.4–1.6 times sensitivity increase. Moreover, all devices exhibit higher sensitivity in the low H_2_ concentration range (0–2000 ppm), with limit of detection at ∼50 ppm. Experimental results align well with simulation trends, with the PMD R50% device with 125 nm Pd coating showing the highest sensitivity at 18.5 kHz/%, and the PMD R70% device with 40 nm Pd coating exhibiting the lowest sensitivity at 6.5 kHz/%. Both simulation and experimental results confirm that R50% devices with thicker Pd coatings demonstrate sensitivity approximately 3 times higher compared to R70% devices with thinner Pd coatings. These findings underscore the significant impact of both piezoelectric stack ratio and Pd coating thickness on H_2_ sensor sensitivity, providing crucial insights for better PMD sensor design.

**Figure 8:**
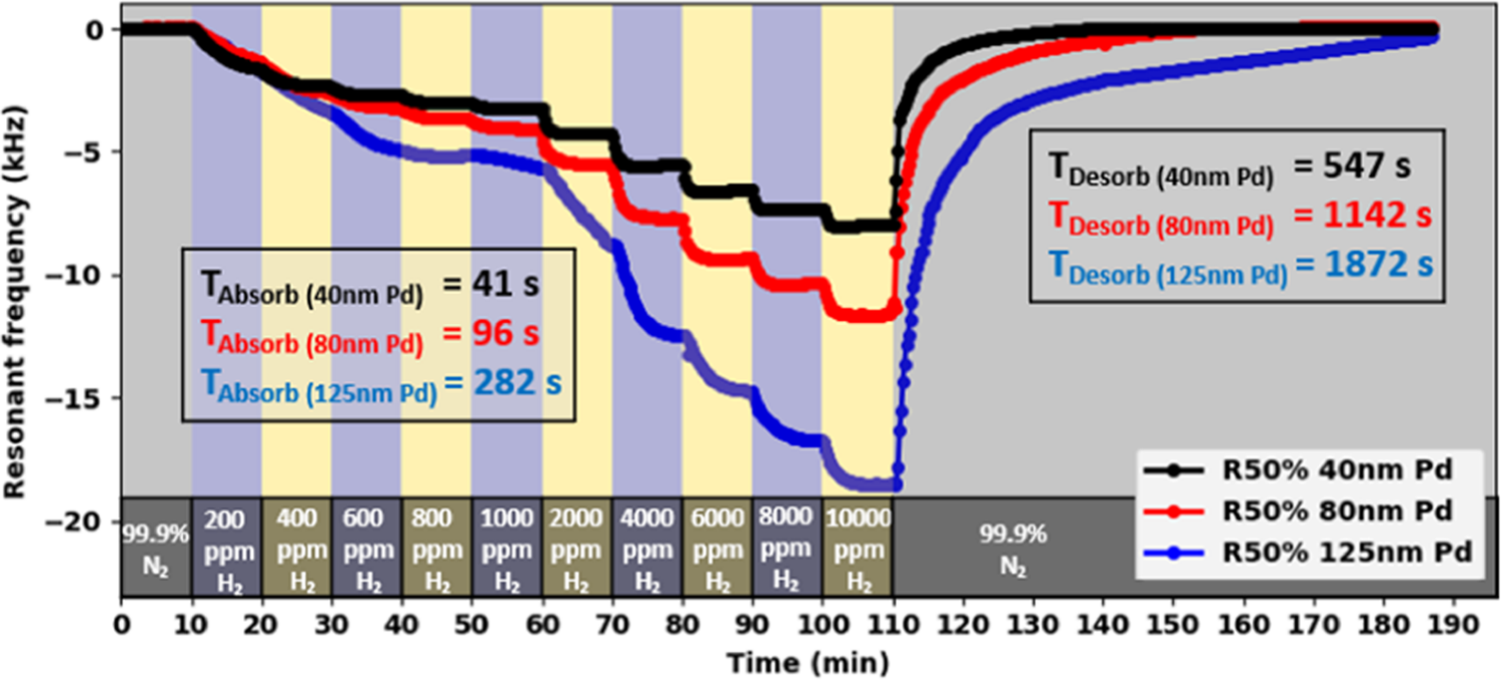
H_2_ response time characterization for PMD R50% 40–125 nm devices.

Figure 8 illustrates the response time characteristics of PMD H_2_ sensors with Pd coating thicknesses ranging from 40 to 125 nm. The 40 nm Pd coating device demonstrates rapid performance, with an average 90% absorption response time of only 41 s and a 90% desorption response time of 547 s. As the thickness of the Pd sensing layer increases, a counterbalancing effect emerges: while response sensitivity improves, both absorption and desorption response times are extended. This relationship can be attributed to the properties of thicker Pd films, which offer larger H_2_ storage capacity and higher saturation thresholds. Since H_2_ gas molecule penetration occurs primarily through the surface area, increasing Pd thickness while maintaining the same penetration rate results in longer saturation times. Consequently, thicker Pd films require more time to reach equilibrium during both the absorption and desorption processes, despite their enhanced sensitivity.

**Figure 9:**
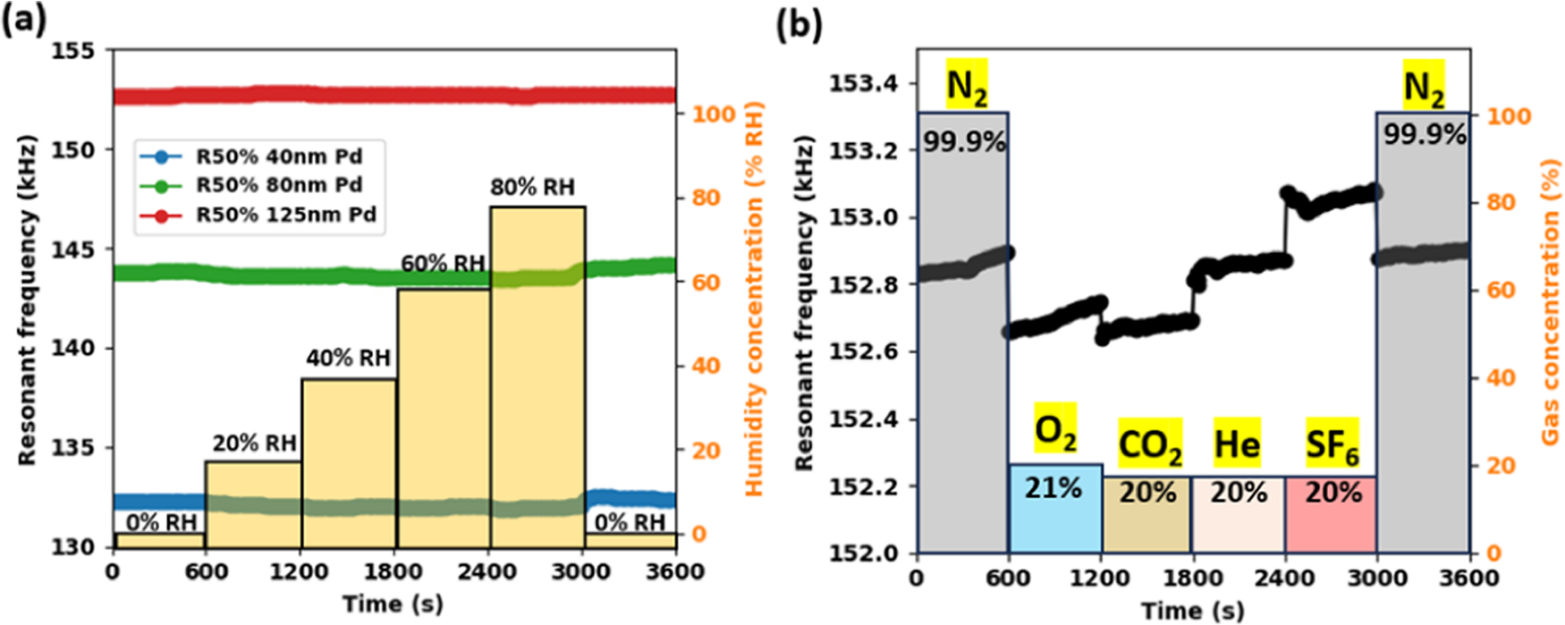
Selectivity characterization for (a) humidity response of PMD R50% 40–125 nm devices. (b) Cross-sensitivity characterization for PMD R50% 125 nm device.

Robustness to humidity is a critical concern for most resonant sensors due to its potential impact on frequency response through mass changes.^25^ To address this, humidity response tests were conducted on PMD R50% devices, with the results presented in Figure 9a. Remarkably, the frequency shifts for all devices remained within ±0.26 kHz across a wide humidity range of 0–80% RH, demonstrating robust performance under varying moisture conditions.

Furthermore, cross-sensitivity characterization was performed on the PMD R50% device with a 125 nm Pd coating, as illustrated in Figure 9b. The sensor was sequentially exposed to 99.9% N_2_, 21% O_2_, 20% CO_2_, 20% He, 20% SF_6_, and back to 99.9% N_2_ gas, with each exposure lasting 10 min. Throughout the 36 min continuous test, the observed frequency shift remained below ±0.25 kHz, indicating excellent selectivity toward H_2_ and minimal interference from other common gases. These results underscore the PMD sensor’s resilience to humidity variations and its high specificity for H_2_ detection, crucial attributes for reliable operation in diverse environmental conditions.

Table 2 summarizes the comparison between the PMD H_2_ sensor developed in this work and the existing resonant H_2_ sensors. Unlike other resonant frequency-based sensors that rely on the Sauerbrey mass-loading equation, where sensitivity is primarily dependent on working frequency, the PMD sensor operates on a stress-based mechanism. Despite functioning at a significantly lower resonant frequency of 150 kHz (1000 times lower than SAW sensors), the PMD sensor achieves a similar level absolute sensitivity of 18.5 kHz/%. Significantly, the FOM for the PMD sensor reaches 10^5^, substantially surpassing other H_2_ resonant sensors. With a compact lateral length of only 600 μm, similar to FBAR dimensions, the PMD sensor is well suited for IC integration. While the response time is currently determined by the sensing material, it offers potential for further optimization through Pd alloy and nano surface structure design.^13−15^

**Table 2:** Comparison of PMD H_2_ Sensor with Existing Resonant H_2_ Sensors

based resonator types	sensing materials	working frequency (MHz)	detect range (%)	sensitivity (kHz/%)	*T*_absorb_/*T*_desorb_ (s)	FOM (ppm/%)	lateral size (μm)	signal quality	ref
FBAR	Pd	2230	0–2	5000	40/70	2.2 × 10^3^	500	high *Q*	(7)
FBAR	ZnO	2390	0–3	6100	20/40	2.6 × 10^3^	500	high *Q*	(8)
SAW	Pd/Cu	150	0–1	1.5	4/4	1.0 × 10^1^	3200	low *Q*	(9)
SAW	Pt/ZnO	129	0–1	55	60/120	4.3 × 10^2^	6000	low *Q*	(10)
QCM	g-C_3_N_4_	9	2–20	2.5 × 10^–3^	200/250	2.8 × 10^–1^	5000	mid *Q*	(11)
QCM	Pd	165	0–0.025	∼5.3	∼29/-	3.2 × 10^1^	1800	mid *Q*	(12)
PMD	Pd (40 nm)	0.13	0–1	8.0	41/547	6.2 × 10^4^	600	mid *Q*	this work
	Pd (125 nm)	0.15		18.5	282/1872	1.2 × 10^5^			

## Conclusions

This work presents a novel PMD H_2_ sensor that significantly advances the field of resonant frequency-based H_2_ sensing. By uniquely integrating a PMD resonator with a Pd sensing layer, we have developed a sensor that operates on a stress-based mechanism, diverging from mass-loading principles. This innovative approach enables the sensor to achieve an unprecedented FOM exceeding 1 × 10^4^, substantially outperforming existing resonant H_2_ sensors. Despite operating at a relatively low frequency of 150 kHz, the PMD sensor demonstrates a remarkable sensitivity of 18.5 kHz/% H_2_, comparable to high-frequency SAW sensors. With a lateral span of just 600 μm, the sensor exhibits a form factor conducive to facile IC integration. Comprehensive characterization, including impedance spectrum analysis, H_2_ sensitivity measurements, and cross-sensitivity tests, confirms the sensor’s excellent performance, with a low limit of detection of 50 ppm and minimal interference from humidity and other gases. While the response time, determined by Pd thickness, presents a trade-off with sensitivity, it offers the potential for further optimization through material engineering. These results underscore the PMD sensor’s potential to revolutionize H_2_ detection across various applications, combining high sensitivity, compact size, and robust performance in a single device.
